# IL-1β increases asporin expression via the NF-κB p65 pathway in nucleus pulposus cells during intervertebral disc degeneration

**DOI:** 10.1038/s41598-017-04384-3

**Published:** 2017-06-23

**Authors:** Shengjie Wang, Chao Liu, Zhongyi Sun, Peng Yan, He Liang, Kai Huang, Changwei Li, Jiwei Tian

**Affiliations:** 1 Department of Orthopedics, Shanghai General Hospital, Shanghai Jiao Tong University School of Medicine, 100, Haining Road, Shanghai, 200080 People’s Republic of China; 2 Shanghai Key Laboratory for Prevention and Treatment of Bone and Joint Diseases with Integrated Chinese-Western Medicine, Shanghai Institute of Traumatology and Orthopedics, Ruijin Hospital, Shanghai Jiaotong University School of Medicine, 197 Ruijin 2nd Road, Shanghai, 200025 People’s Republic of China; 3 Department of Orthopedics, Songjiang District Central Hospital of Shanghai, 746, middle-Zhongshan Road, Shanghai, 201600 People’s Republic of China

## Abstract

Disc degeneration (DD) is a multifaceted chronic process that alters the structure and function of intervertebral discs. The pathophysiology of degeneration is not completely understood, but the consensus is that changes in genes encoding extracellular matrix (ECM) proteins in the disc are the leading factors contributing to DD. Asporin is an ECM protein that has been shown to be increased in degenerated intervertebral discs, but little is known about how asporin is regulated during DD. In exploring the intricate mechanism, we confirmed that asporin was abundantly increased in patients’ degenerated nucleus pulposus. Consistently, the increased asporin expression with degeneration was also proved by rabbit intervertebral disc degeneration (IDD) model. Mechanistically, IL-1β upregulated asporin expression by activating the p65 pathway in human nucleus pulposus cells. Furthermore, p65 mediated asporin expression by binding to −41/−31 bp on *asporin* promoter. Functionally, asporin was the intermediator of IL-1β-inhibited aggrecan and collagen Π expression and played a negative role in TGF-β-induced aggrecan and collagen Π formation in human nucleus pulposus cells. Therefore, identifying asporin as a negative regulator of aggrecan and collagen Π and elucidating its induction mechanisms in human nucleus pulposus cells provides new insight for asporin induction during IDD.

## Introduction

Disc degeneration (DD) is a multifaceted chronic process that can lead to herniation, radiculopathy, myelopathy, spinal stenosis, and degenerative spondylolisthesis, resulting in acute or chronic pain that alters the structure and function of the intervertebral discs and can lead to painful conditions^1^. It is a common and serious problem worldwide that affects 40% of individuals younger than 30 years old and more than 90% of those older than 50 years old^2^. Thus, the development of innovative therapies is eagerly awaited by the millions of patients suffering from this condition.

The etiology of DD is not completely understood, but the consensus is that degeneration begins as early as the second decade of life^3^. In the early stages of degeneration, cells attempt to repair the degeneration by making collagens and proteoglycans^4^, but as the degeneration proceeds, synthesis slows down, and the disc components are broken down^5^. A decrease in collagen and proteoglycan synthesis leads to a loss of water, height, and hydrostatic pressure. Over time, this loss can cause decreased flexibility and strength^1^. Thus, the genes encoding extracellular matrix (ECM) proteins expressed in the nucleus pulposus (inner structure) and annulus fibrosus (outer layer) of the disc, such as type Π collagen, type IX collagen, aggrecan, and cartilage intermediate layer protein (CILP), have been shown to be risk factors for disc degeneration^6–9^.

Recently, an extracellular matrix protein, asporin (ASPN), was shown to be upregulated in articular cartilage during osteoarthritis, a progressive degenerative joint disease. Furthermore, asporin was found to negatively regulate the differentiation of chondrocytes and the expression of type Π collagen and aggrecan by inhibiting transforming growth factor β (TGF-β) signaling^10^. Asporin belongs to the small leucine-rich proteoglycan (SLRP) family. Its N-terminus contains a unique aspartate (D) residue repeat, which is a polymorphic region in the gene with alleles that encode D repeats ranging from 9–20 residues. The D14 polymorphism, with 14 D residues, has been identified as a risk allele for OA^10^. In addition to being involved in the pathogenesis of osteoarthritis, asporin has been reported to be associated with disc degeneration. It has been found that asporin expression in vertebral discs increases with disc degeneration^6, 11^, and a meta-analysis showed that Chinese and Japanese individuals harboring a D14 allele had higher risk of lumber disc degeneration^6^. However, little is known about how asporin is regulated and becomes involved in the pathogenesis of intervertebral disc degeneration.

Given that the prevalence of intervertebral disc degeneration requires the development of innovative therapies and that asporin was reported to be associated with the disc degeneration, we set out to investigate whether asporin is expressed in degenerated discs and to determine if asporin exhibits a significant functional relevance in this system. Our findings uncovered a vital detrimental role of asporin in promoting disc degeneration and delineated a previously unknown intrinsic regulatory mechanism.

## Results

### Asporin was upregulated in the nucleus pulposus of degenerated intervertebral discs

**Figure 1:**
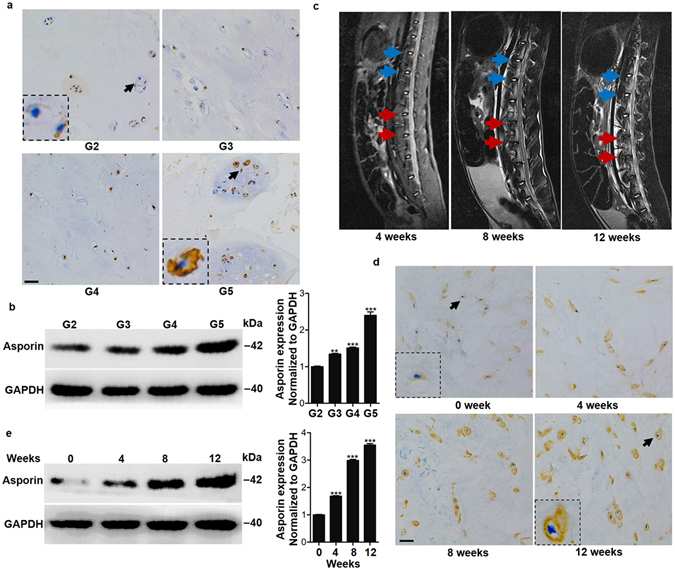
Asporin was upregulated in nucleus pulposus tissues of degenerated intervertebral discs. (**a**) Immunohistochemistry staining of asporin in nucleus pulposus tissues of patients’ intervertebral discs with different grades of degeneration. (**b**) Western blot analysis of asporin expression in nucleus pulposus tissues of patients’ intervertebral discs with different grades of degeneration. Each point represents samples from 6 patients with different grades of degeneration pooled together. (**c**) Magnetic resonance imaging of the intervertebral disc at different time points post-annulus needle puncture. Blue arrows indicate non-puncture and red arrows indicate annulus needle puncture. (**d**) Immunohistochemistry staining of asporin in nucleus pulposus tissues of rabbit intervertebral discs at different time points after inducing intervertebral disc degeneration. (**e**) Western blot analysis of asporin expression in nucleus pulposus tissues of rabbit intervertebral discs at different time points after inducing intervertebral disc degeneration. Each time point represents samples from 4 animals pooled together. **P < *0.01*, ***P < *0.001*. *P*-values were analyzed by one-way ANOVA. Scale bars represent 10 μm. All data are representative of three independent experiments. Uncropped images of the blots for Fig. 1b,e are shown in supplementary Figure 3.

Although increased asporin has been reported during disc degeneration, the intricate underlying mechanism by which asporin becomes involved in the pathogenesis of disc degeneration remains largely unknown. To explore the role of asporin in disc degeneration, we first evaluated the expression of asporin in the nucleus pulposus of patients with intervertebral disc degeneration. Immunohistochemical (IHC) staining showed that more degenerated human discs (Pfirrmann grade 5) expressed higher levels of asporin than the less degenerated (grades 2, 3 and 4) discs. As the severity of disc degradation increased, the asporin staining intensity became stronger (Fig. 1a). Consistent with the IHC result, the enhanced asporin expression with increased degeneration severity was further shown by western blot analysis (Fig. 1b). To further confirm that the increase in nucleus pulposus asporin expression had a causal relationship with disc degeneration, we used a rabbit intervertebral disc degeneration model^12^ and evaluated asporin expression. The annulus needle puncture (red arrows) induced significantly more disc degeneration than in normal controls (non-puncture, blue arrows), and the degeneration severity increased with time (Fig. 1c). Asporin expression was abundantly upregulated with disc degeneration, which was demonstrated by IHC staining and western blot analysis (Fig. 1d,e). In addition, IHC staining and western blot analysis results revealed that needle puncture injury induced time-dependent expression of asporin in the annulus fibrosus (Supplementary Figure 1a) and cartilage endplate (Supplementary Figure 1b,c). Taken together, these data demonstrated that asporin is upregulated in the nucleus pulposus during intervertebral disc degeneration.

### IL-1β upregulated asporin expression in human cells isolated from nucleus pulposus tissue

**Figure 2:**
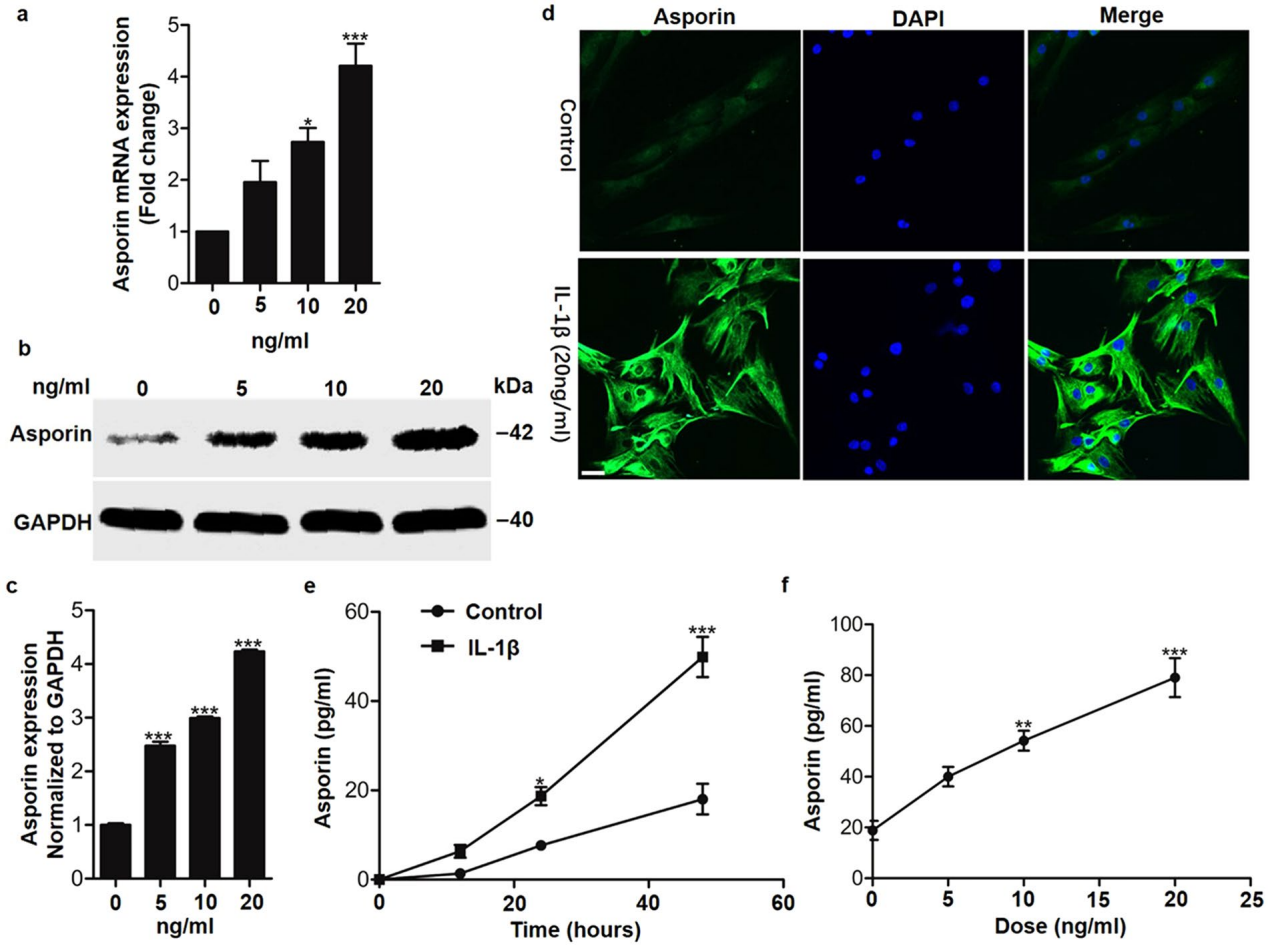
IL-1β upregulated asporin expression in human cells isolated from nucleus pulposus tissues. (**a**) Quantification of asporin mRNA expression in human nucleus pulposus cells treated with different doses of IL-1β. (**b** and **c**) Western blot analysis of asporin expression in human nucleus pulposus cells treated with different doses of IL-1β. (**d**) Immunofluorescence analysis of asporin in human nucleus pulposus cells induced by 10 ng/ml IL-1β. (**e** and **f**) Quantification of asporin detected by ELISA in the culture supernatant of human nucleus pulposus cells induced by IL-1β. Scale bars represent 10 μm. *P < *0.05*, **P < *0.01*, ***P < *0.001*. *P*-values were analyzed by one-way ANOVA in a & c and two-way ANOVA in (**e** and **f**). All data are representative of three independent experiments and are means ± SEM. Uncropped images of the blots for Fig. 2b are shown in supplementary Figure 3.

Having observed that asporin was upregulated during intervertebral degeneration, we next sought to explore the molecular mechanisms involved in the induction of asporin. Since proinflammatory cytokines, especially TNF-α and IL-1β, are principally associated with the progression of IDD^13–15^, we hypothesized that IL-1β might take part in the pathogenesis of IDD through asporin. First, we detected asporin expression induced by IL-1β in in primary human nucleus pulposus cells. The mRNA expression detected by real time RT-PCR and protein expression detected by western blot all showed that IL-1β induced asporin in a dose-dependent manner (Fig. 2a–c). Furthermore, the increased asporin expression induced by IL-1β was demonstrated by immunofluorescence analysis (Fig. 2d). Consistent with the finding that asporin is a secreted protein^10^, we detected an increased concentratin of asporin in human nucleus pulposus in respond to IL-1β stimulation (Fig. 2e,f), and the concentration could reach approximately 80 pg/ml after 48 hours of 20 ng/ml IL-1β stimulation (Fig. 2e). Taken together, these data demonstrated that IL-1β can work as a stimulus to upregulate asporin expression in human nucleus pulposus cells.

### IL-1β upregulated asporin expression via the NF-κB p65 pathway

**Figure 3:**
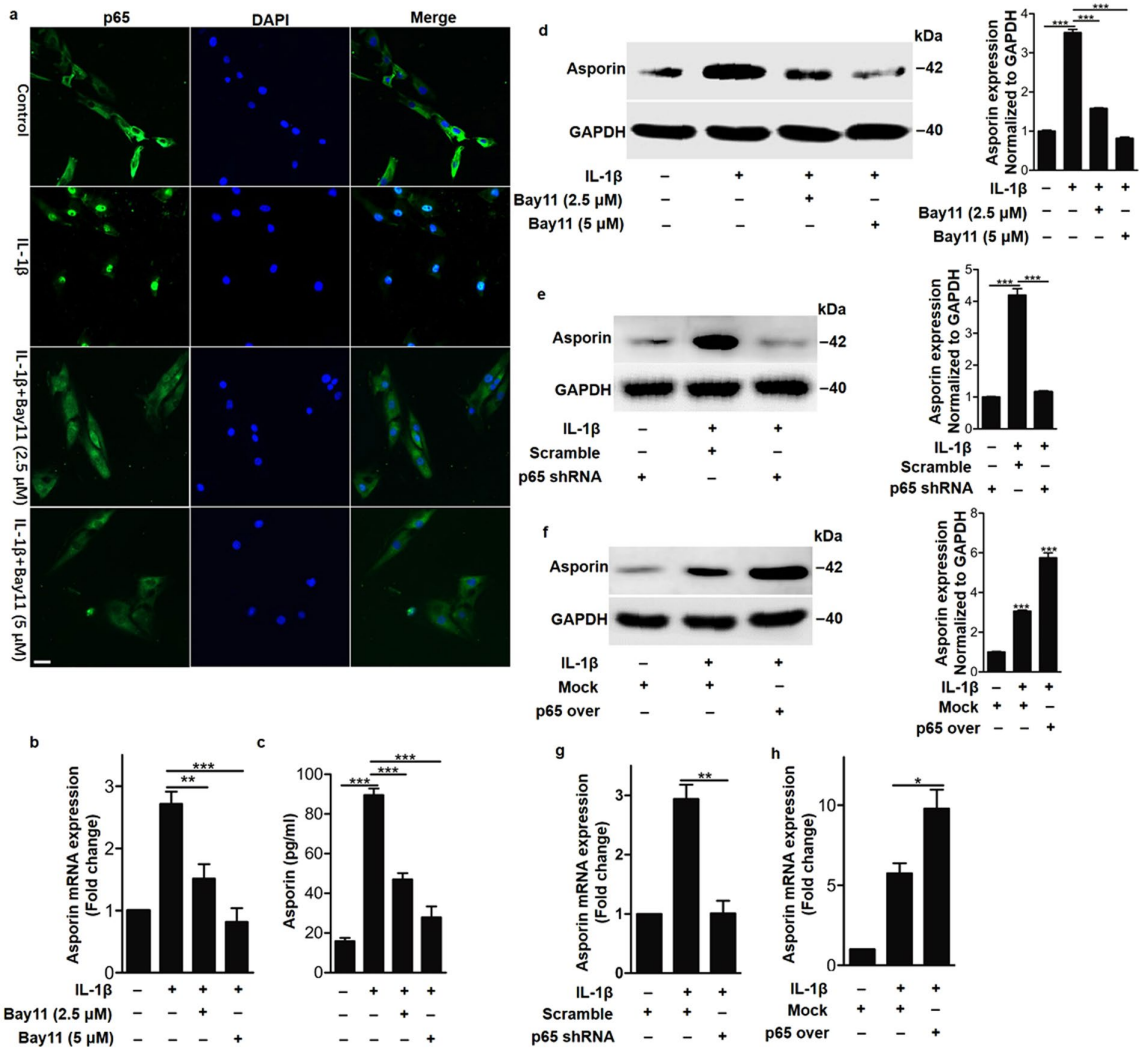
IL-1β upregulated asporin expression in human nucleus pulposus cells via the NF-κB p65 pathway. (**a**) Immunofluorescence analysis of p65 in human nucleus pulposus cells induced by 10 ng/ml IL-1β with or without the p65 inhibitor Bay 11. (**b**) Quantification of asporin mRNA expression in human nucleus pulposus cells treated with 10 ng/ml IL-1β with or without Bay 11. (**c**) Quantification of asporin secretion in the culture supernatant of human nucleus pulposus cells treated with 10 ng/ml IL-1β with or without Bay 11. (**d**) Western blot analysis of asporin expression in human nucleus pulposus cells treated with 10 ng/ml IL-1β with or without Bay 11. (**e** and **g**) Western blot and real time RT-PCR analysis of asporin expression in human nucleus pulposus cells treated with 10 ng/ml IL-1β in the presence or absence of p65 shRNA. (**f–h**) Western blot and real time RT-PCR analysis of asporin expression in human nucleus pulposus cells induced by 10 ng/ml IL-1β with or without p65 overexpression. p65 over means p65 overexpression; Scramble and Mock represent the negative controls of p65 shRNA and p65 overexpression. *P < 0.05, **P < *0.01*, ***P < *0.001*. *P*-values were analyzed by one-way ANOVA. All data are representative of three independent experiments and are means ± SEM. Uncropped images of the blots for Fig. 3d–f are shown in supplementary Figure 3.

Having observed that IL-1β enhanced asporin expression in human nucleus pulposus cells, we next sought to investigate the intrinsic mechanisms by which IL-1β regulates asporin expression. IL-1β increases the activity of nuclear factor κB (NF-κB), a transcription factor associated with various diseases^16^. Furthermore, using GeneChip array techniques (GeneChip(R) Human X3 P Array) to compare gene expression between degenerated discs and non-degenerated discs and predicting relevant signaling pathways using the KEGG (Kyoto Encyclopedia of Genes and Genomes) database^17^, we found that the NF-κB pathway was significantly upregulated in human cells isolated from degenerated nucleus pulposus tissue compared to cells isolated from non-degenerated tissue (Supplementary Figure 2a). We predicted that IL-1β might increase asporin expression via the NF-κB p65 pathway. To assess the function of p65 during this process, we first detected p65 activation induced by IL-1β in human nucleus pulposus cells. Working as a nuclear factor, p65 exerts transcriptional activity by translocating the nucleus to induce the expression of its target genes^18, 19^. We first detected p65 nuclear translocation in the presence or absence of IL-1β in human nucleus pulposus cells. The immunofluorescence analysis revealed that p65 was preferentially distributed in the cytoplasm rather than in the nucleus in the control group, but under IL-1β stimulation, most p65 translocated to the nucleus (Fig. 3a). Furthermore, p65 translocation was abundantly inhibited by the NF-κB p65 pathway-specific inhibitor BAY 11 (Fig. 3a), which indicates that IL-1β could activate the NF-κB p65 pathway. Next, we detected asporin expression under IL-1β with or without p65 activation. The mRNA expression detected by real time PCR and the protein expression detected by western blot all demonstrated that p65 was a crucial adaptor for IL-1β induced asporin, as the upregulated asporin expression induced by IL-1β was significantly dampened by the p65 inhibitor Bay 11 (Fig. 3b–d). To further demonstrate the function of p65 in IL-1β-induced asporin expression, we conducted a loss-of-function experiment in which we knocked down p65 by shRNA. Similar to the p65 expression results, we found that IL-1β could not upregulate asporin expression after p65 depletion (Fig. 3e,g, supplementary Figure 2b). Lastly, the crucial role of p65 was further demonstrated by the gain of function in which we overexpressed p65. The results showed that p65 overexpression significantly enhanced IL-1β-induced asporin expression (Fig. 3f,h, supplementary Figure 2c). Taken together, these data demonstrated that IL-1β upregulates asporin expression via the NF-κB p65 pathway.

### The asporin promoter contained p65 binding sites

**Figure 4:**
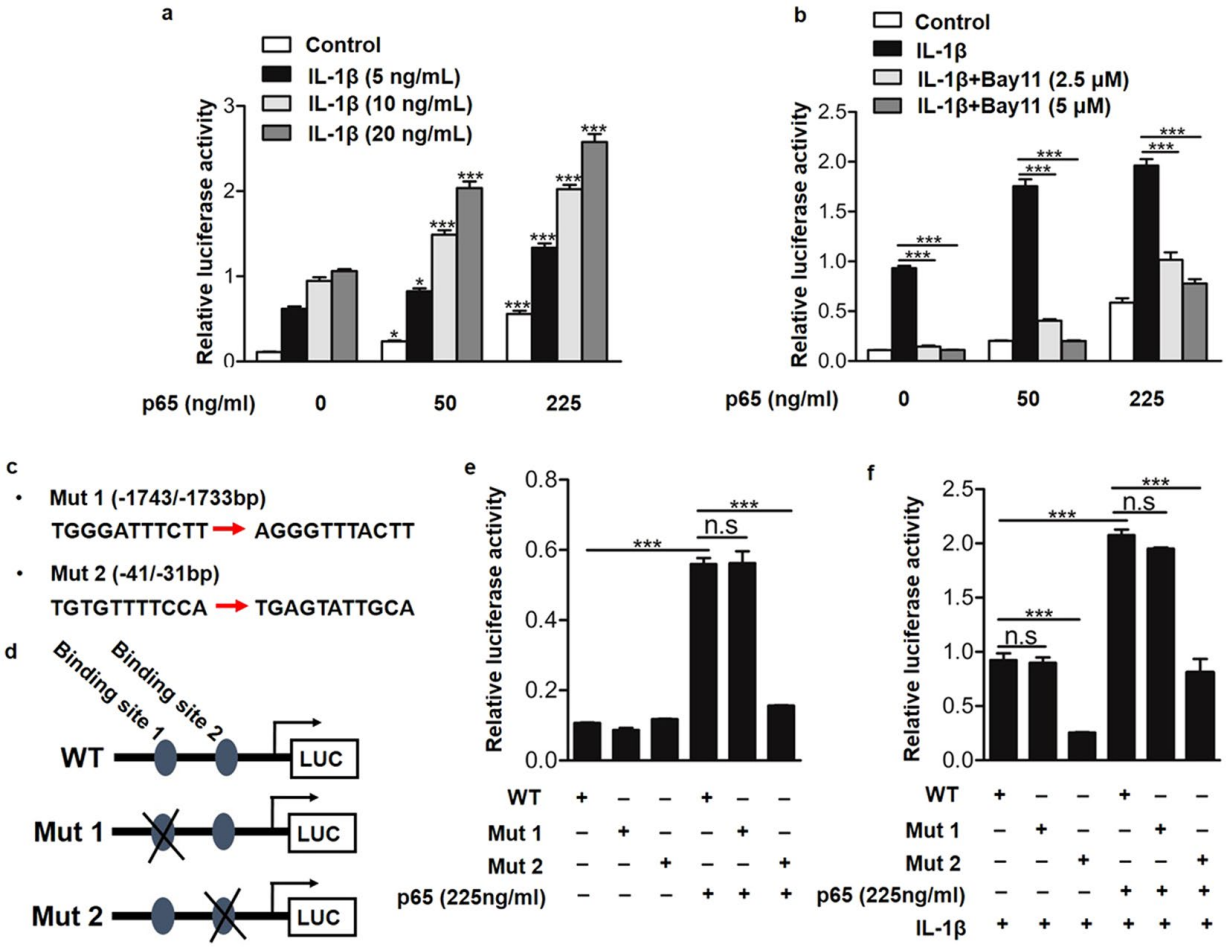
p65-mediated asporin promoter activity directly in human nucleus pulposus cells. (**a**) Asporin promoter activity detected by dual-luciferase reporter gene assay system in the presence of different doses of p65 and/or IL-1β. (**b**) Asporin promoter activity detected by dual-luciferase reporter gene assay system in the presence of different doses of p65/Bay 11 and/or 10 ng/ml IL-1β. (**c**) The specific sequences of the two putative p65 binding sites on the asporin promoter. (**d**) The schematic of the wild-type and mutant human asporin promoter constructs. (**e** and **f**) Asporin promoter activity induced by different doses of p65 and/or 10 ng/ml IL-1β with or without putative p65 binding sites mutation on asporin promoter. *P < *0.05*, ***P < *0.001*. *P* values were analyzed by one-way ANOVA. All data are representative of three independent experiments and are means ± SEM.

As a transcription factor, p65 exerts biological activity by translocation to the nucleus and binding to specific gene promoters^20^. Furthermore, we observed that NF-κB activation was essential for IL-1β-induced asporin expression. We hypothesized that activated p65 might stimulate the production of asporin by directly binding at its promoter region. To test our hypothesis, we first used the dual-luciferase reporter gene assay system to detect the promoter activity of asporin induced by p65. The results revealed that p65 overexpression increased asporin promoter activity in a dose-dependent manner. Given that IL-1β upregulated asporin expression via the p65 pathway (Fig. 2), we then assessed whether IL-1β enhanced asporin promoter activity induced by p65 overexpression. After 6 hours of p65 overexpression via plasmid transfection, the human cells isolated from nucleus pulposus tissue were treated with different doses of IL-1β. The results showed that IL-1β and p65 overexpression had a synergistic effect in increasing asporin promoter activity (Fig. 4a). However, this increased promoter activity was markedly dampened by the p65 inhibitor Bay 11 (Fig. 4b). Having observed that p65 enhanced asporin promoter activity directly, we next sought to identify the asporin promoter region at which p65 would bind. An analysis of the human asporin promoter using the JASPAR core database^21^ revealed the presence of two putative p65 binding sites, −1743/−1733 and −41/−31 bp, whose specific sequences are shown in bold in Fig. 4c. To further examine if the predicted binding sites are necessary for promoter regulation by p65, we performed site-directed mutagenesis to mutate the binding sites individually (Fig. 4c). Figure 4d shows a schematic of the wild-type and mutant human asporin promoter constructs. Cells were transfected with these constructs, and luciferase activity was measured following co-transfection with p65. The results showed that compared with the wild-type promoter, the −41/−31 bp mutation resulted in a decrease in asporin promoter activity (Fig. 4e). The mutation of the −1743/−1733 bp region had no effect on p65-induced asporin reporter activity (Fig. 4e). In addition, the presence of a p65 binding site at −41/−31 bp on the asporin promoter was further supported by IL-1β, as IL-1β increased asporin promoter activity that was abundantly dampened after −41/−31 bp mutation (Fig. 4f). Together, these results suggest that p65 regulates asporin promoter activity by binding to the −41/−31 bp site on the asporin promoter.

### IL-1β upregulated asporin to regulate aggrecan and type Π collagen expression

**Figure 5:**
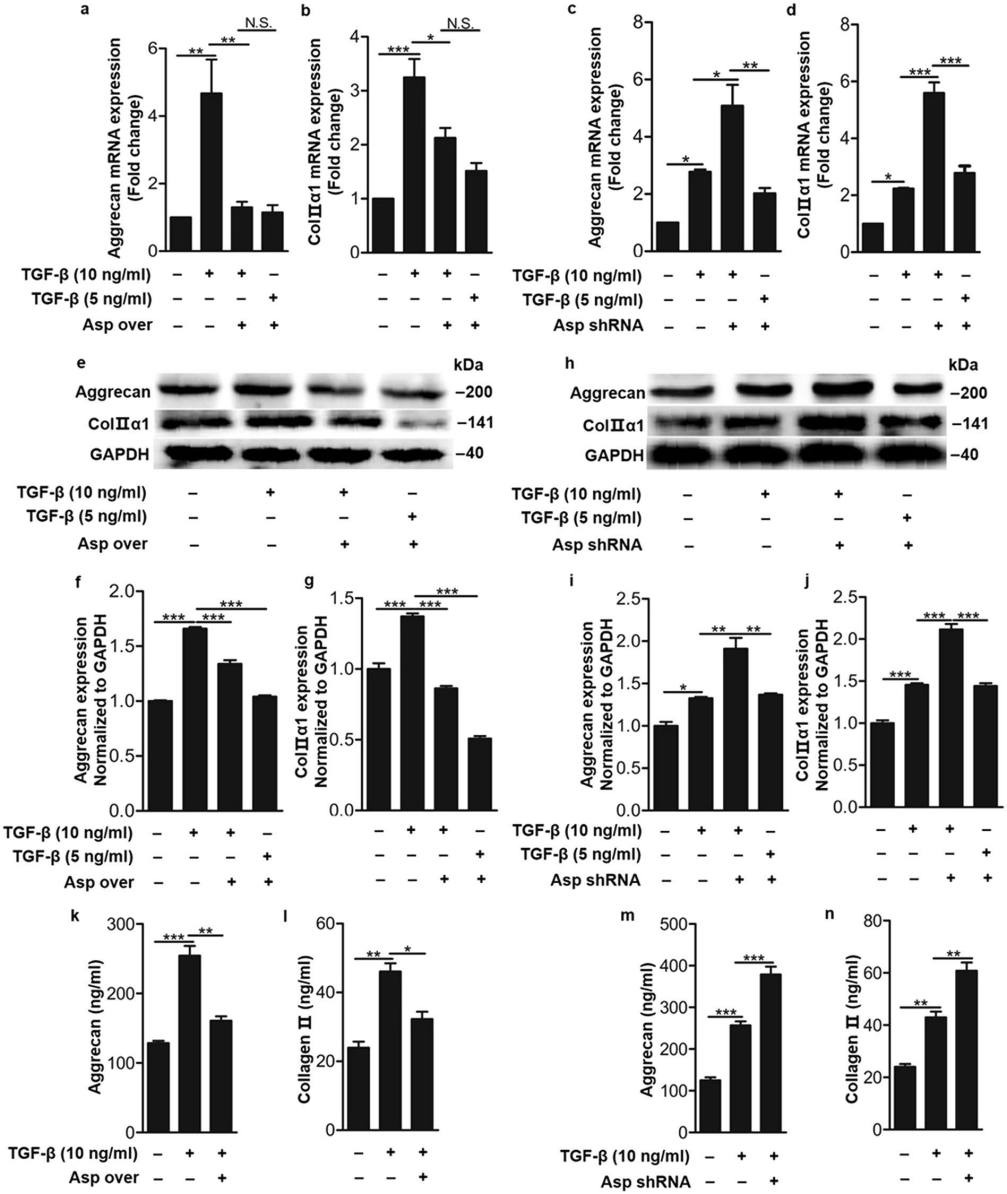
Asporin mediated TGF-β-induced aggrecan and type Π collagen synthesis and secretion in human nucleus pulposus cells. (**a, b, e–g**) Aggrecan and type Π collagen expression detected by quantitative real time RT-PCR and western blot in human nucleus pulposus cells induced by TGF-β with or without asporin overexpression. “over” means overexpression. (**k** and **l**) Aggrecan and type Π collagen secretion detected by ELISA in culture medium of human nucleus pulposus cells induced by TGF-β with or without asporin overexpression (**c, d, h–j**) Aggrecan and type Π collagen expression detected by quantitative real time RT-PCR and western blot in human nucleus pulposus cells induced by TGF-β in the presence or absence of asporin shRNA. (**m** and **n**) Aggrecan and type Π collagen secretion detected by ELISA in culture medium of human nucleus pulposus cells induced by TGF-β in the presence or absence of asporin shRNA. *P < *0.05*, **P < *0.01*, ***P < *0.001*. *P*-values were analyzed by one-way ANOVA. All data are representative of three independent experiments and are means ± SEM. Uncropped images of the blots for Fig. 5e and h are shown in supplementary Figure 3.

**Figure 6:**
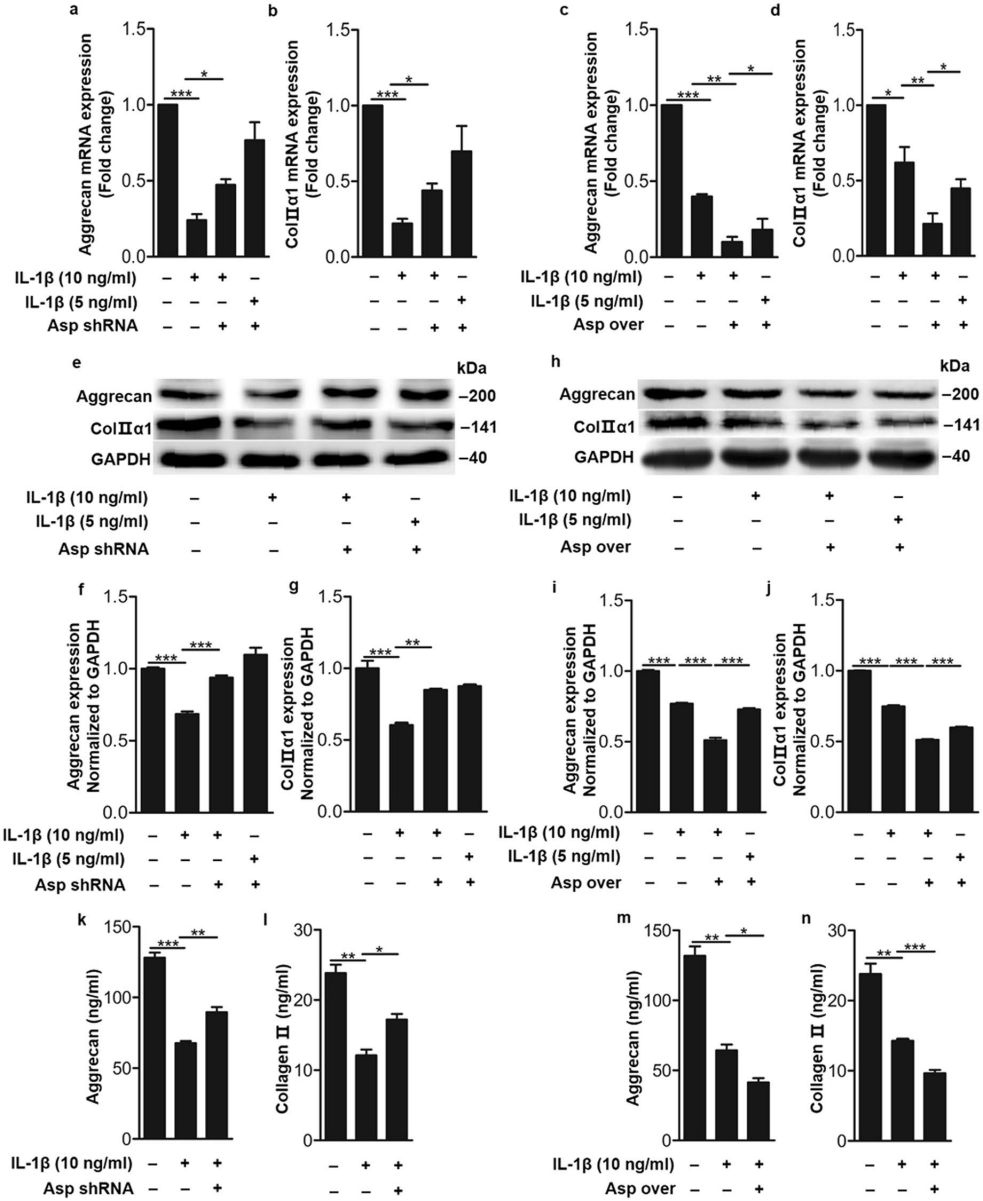
Asporin mediated IL-1β-dampened aggrecan and type Π collagen synthesis and secretion in human nucleus pulposus cells. (**a** and **b**, **e–g**) Aggrecan and type Π collagen expression detected by quantitative real time RT-PCR and western blot in human nucleus pulposus cells induced by IL-1β in the presence or absence of asporin shRNA. (**k** and **l**) Aggrecan and type Π collagen secretion detected by ELISA in human nucleus pulposus cells induced by IL-1β in the presence or absence of asporin shRNA. (**c** and **d**, **h–j**) Aggrecan and type Π collagen expression detected by quantitative real time RT-PCR and western blot in human nucleus pulposus cells induced by IL-1β with or without asporin overexpression. Over means overexpression. (**m** and **n**) Aggrecan and type Π collagen secretion detected by ELISA in human nucleus pulposus cells induced by IL-1β with or without asporin overexpression. *P < *0.05*, **P < *0.01*, ***P < *0.001*. *P*-values were analyzed by one-way ANOVA. All data are representative of three independent experiments and are means ± SEM. Uncropped images of the blots for Fig. 6e,h are shown in supplementary Figure 3.

It is known that asporin inhibits chondrogenesis in knee and hip joint cartilage by dampening TGF-β–induced aggrecan and type Π collagen expression^10^. Consistent with these observations, we found that asporin overexpression significantly dampened TGF-β-increased aggrecan and type Π collagen expression and secretion in human cells isolated from nucleus pulposus tissues (Fig. 5a,b,e–g,k and l supplementary Figure 2e). Asporin knockdown significantly enhanced TGF-β-induced aggrecan and type Π collagen expression and secretion (Fig. 5c,d,h–j,m and n, supplementary Figure 2d). Furthermore, the loss-of-function experiment showed that after asporin was knocked down by shRNA, the decreased expression levels of aggrecan and type Π collagen expression and secretion mediated by IL-1β were significantly restored (Fig. 6g,b,e–g,k and l). However, asporin overexpression in human nucleus pulposus cells substantially enhanced the inhibitory aggrecan and type Π collagen expression and secretion by IL-1β (Fig. 6c,d,h–j,m and n). Taken together, these data demonstrated that asporin dampens aggrecan and type Π collagen expression in human nucleus pulposus cells.

## Discussion

Disc degeneration is a chronic and polygenic disease that alters the structure and function of the intervertebral discs and can lead to painful conditions^1^. The pathophysiology of degeneration is not well understood, but previous studies suggested that the changes in the sequences of genes encoding extracellular matrix (ECM) proteins expressed in the nucleus pulposus and annulus fibrosus of the disc may be important contributing factors leading to an increased risk for disc degeneration^6^. Asporin, an extracellular matrix protein, has been shown to be upregulated in degenerated intervertebral discs, but its function and intrinsic expression mechanisms still need further study. Here, we confirmed that asporin expression in nucleus pulposus tissues increased with disc degeneration. Mechanistically, NF-κB p65 translocated to the nucleus and regulated asporin expression by binding to the −41/−31 bp site on the asporin promoter in human nucleus pulposus cells. Moreover, IL-1β increased asporin expression in isolated human cells from nucleus pulposus tissues via the p65 pathway. Functionally, asporin was essential for IL-1β inhibited aggrecan and type Π collagen expression and played a negative role in TGF-β-induced aggrecan and type Π collagen formation *in vitro*. Therefore, the identification of asporin as a negative regulator of aggrecan and type Π collagen, as well as the elucidation of the mechanisms by which it is induced in human nucleus pulposus cells, may provide potential immune-based anti-disc degeneration therapies by targeting asporin expression.

Asporin is a recently identified ECM protein that contains a unique D repeat in its N-terminal region^10, 22^. It is a member of the family of small leucine-rich proteoglycans and is most closely related to decorin and biglycan^23^. Previous studies revealed that asporin contains a unique aspartate (D) residue repeat in the N terminus, which is a polymorphic region in the gene with alleles that encode D repeats ranging from 9–20 residues^22^. The D14 polymorphism with 14 D residues has been identified as a risk allele for osteoarthritis^10^ and has also been shown to be associated with IDD^6, 11^. Asporin mRNA has been reported to be highly expressed in osteoarthritis articular cartilage^22^. Functional studies demonstrated that ASPN inhibited *in vitro* chondrogenesis and the expression of *ColΠα1* and *Agc1* through the inhibition of TGF-β signaling^10^. Since osteoarthritis and intervertebral disc degeneration are both degenerative diseases of skeletal joint regions, and because many of the genes expressed in cartilage are also expressed in the intervertebral disc, we tested whether asporin could mediate aggrecan and collagen Π expression in human nucleus pulposus cells during disc degeneration.

It has been shown that asporin was increased in both Asian and Caucasian individuals with disc degeneration. In contrast to the present results and previous findings^6^ that asporin was upregulated in the degenerated discs of Asian subjects, including the nucleus pulposus and annulus fibrosus, compared with the less degenerated discs, Gruber *et al*. found greater immunolocalization of asporin in the outer annulus than in the inner annulus, whereas localization was rare in the nucleus pulposus of the degenerated discs of Caucasians^11^. Whether the discrepancy is due to racial differences or other reasons still need for further investigation.

Despite the fact that the etiology of IDD is poorly understood, it is clear that IDD is not only due to an imbalance between the anabolism and catabolism of the extracellular matrix (ECM) but also results from changes in cell phenotype that can have an impact on ECM formation^24^, leading to large structural changes therein^25^. Increasing data suggest that inflammatory cytokines, especially IL-1β produced by nucleus pulposus cells within the IVD during degeneration, are responsible for matrix degradation and induction of lower back pain^13, 26, 27^. IL-1β has also been shown to regulate a plethora of events linked to IVD degeneration, such as increased matrix degradation, decreased matrix synthesis, increased proinflammatory cytokine production and the increased production of neurotrophic and angiogenic factors^28^. Furthermore, IL-1 receptor antagonist-deficient mice exhibit spontaneous disc degeneration, which, together with the increased risk of low back pain in patients with IL-1β polymorphisms, suggests a major role for IL-1β in the pathogenesis of disc degeneration^28^. In agreement with these observations, our findings revealed that IL-1β could also mediate disc degeneration by regulating asporin expression. Moreover, IL-1β upregulated asporin expression through the NF-κB p65 pathway.

Nuclear factor kappa B (NF-κB) is a family of transcription factors that plays a central role in mediating cellular responses to damage, stress, and inflammation^29^. It has been reported that NF-κB plays a crucial role in the production of inflammatory mediators and catabolic gene expression, such as MMP1, MMP13, ADAMTS-4 and ADAMTS-5 in chondrocytes^30, 31^. It has been reported that NF-κB is an important catabolic pathway in the pathophysiology of IDD^29^. Under basal conditions, NF-κB is localized primarily in the cytoplasm in an inactive state because it is sequestered by the IκB proteins. NF-κB is activated and translocated into the nucleus in response to many different types of stresses, including inflammatory and oxidative stress^32^. Here, we found that NF-κB activation was essential for asporin expression in degenerated intervertebral discs. Furthermore, we found that under IL-1β stimulation, NF-κB translocated into the nucleus and bound to the promoter of asporin. We further found that the −41/−31 bp region was a p65 binding site.

However, we would like to point out some potential limitations of our study. First, although our results revealed that asporin worked as an intermediator in IL-1β-inhibited aggrecan and collagen Π expression by mediating p65 activity, these results were acquired based on data from human nucleus pulposus cells. Whether the same mechanisms are shared in other IVD cell types (annulus fibrosus and cartilaginous endplate) is unknown. Furthermore, the exact induction mechanisms of asporin *in vivo* still require further investigation. Second, the human nucleus pulposus cell cultures were not monitored under conditions of hypoxia, which is physiologically relevant and may have affected cell growth. Third, we did not consider the incubation of human nucleus pulposus cells with ascorbic acid, which is essential for the proper synthesis and secretion of extracellular matrix proteins, such as aggrecan and collagens^33, 34^. Fourth, many pro-inflammatory cytokines are relevant to the pathogenesis of IVD degeneration (IL-1β, IL-6, IL-8, and IL-17)^35^, and stimulation with more pro-inflammatory cytokines other than IL-1β would improve the understanding of asporin expressed in nucleus pulposus cells and its contribution to degeneration. We chose to stimulate the cells with IL-1β because it is associated with IVD injury^36^, and its presence increases with degeneration, which may contribute to matrix breakdown^37^. Finally, needle puncture-induced IVD degeneration is a rapid and acute process^38^ that does not closely mimic the slowly evolving IVD degeneration observed in humans. Therefore, animal models that are more clinically relevant are critical for assessing the function of asporin in disc degeneration.

In conclusion, our study provides evidence that asporin is an important risk factor in intervertebral disc degeneration. The identification of the intrinsic expression mechanisms of asporin provides further understanding of disc degeneration.

## Material and Methods

### Human samples

This study was approved by the Shanghai First People’s Hospital Ethics Committee. All patients and their next of kin signed an informed consent form allowing the researchers to use IVD tissues obtained during spinal surgery. All donor patients underwent spinal fusion surgery, which requires the intervertebral disc to be resected.

Living intervertebral disc specimens were obtained from 31 patients who underwent discectomy for degenerative or traumatic disc disease. Patients were aged between 17 and 48 years old, and the mean age was 31.37 years old. After dissecting the disc space, a rectangular cut around the annulus fibrosus was made with a sharp knife, and, using pituitary forceps, the disc was removed with the annulus and NP en bloc. After confirming the margin of the annulus and nucleus, only the NP was excised for this study to ensure sample homogeneity. According to the Pfirrmann grading scale^39^, the discs were nondegenerative (Pfirrmann < grade III) and degenerative IVD tissue samples. A portion of the tissue was stored in liquid nitrogen for later use, and the other portion was directly sent to the cell culture laboratory super clean bench for primary culture of the nucleus pulposus cells.

### Rabbits

27 New Zealand rabbits were purchased from Shanghai Laboratorial Animal Center, Chinese Academy of Sciences. The animals were housed with free access to water and a standard rat diet in an air-conditioned room with a 12-hour light-dark cycle at 21 °C to 23 °C and 60% relative humidity in the animal facility at Ruijin Hospital, Shanghai Jiao Tong University School of Medicine, China.

### Ethics statement

All human sample acquisitions were approved by the ethics committee of Shanghai First People’s Hospital, Shanghai Jiao Tong University School of Medicine, China, and performed in accordance with the principles of the Declaration of Helsinki. All participants provided written informed consent, which was obtained before enrollment in the study. All animal experiments were performed according to the protocol approved by the Shanghai Jiao Tong University (SJTU) Animal Care and Use Committee and in direct accordance with the Ministry of Science and Technology of the People’s Republic of China Animal Care Guidelines. The protocol was approved by the SJTU Animal Care and Use Committee. All surgeries were performed under anesthesia, and all efforts were made to minimize suffering.

### Reagents

The reagents used in this study were as follows: rabbit anti-human NF-kB p65 monoclonal antibody (Abcam, ab7970, USA), rabbit anti-rabbit NF-kB p65 monoclonal antibody (NOVUS, NBP1–36209, USA), rabbit anti-human asporin monoclonal antibody (Abcam, ab58741, USA), rabbit anti-rabbit asporin monoclonal antibody (Sigma, AV42487, USA), rabbit aggrecan (Santa Cruz, sc-25674, USA), rat collagen Π (Abcam, ab3029, USA), rabbit GAPDH (Abcam, ab181602, USA), Trizol reagent (Invitrogen, USA), reverse transcription kit (TaKaRa, Dalian, China), fluorescence quantification kit (TaKaRa, Dalian, China), BAY11-7082 (Sigma, USA), recombinant human IL-1β (Peprotech, 1202B95R1, USA). recombinant human TGF-β1 (Peprotech, 0216209, USA). Lipofectamine 3000 (life, USA), Dual-Luciferase Reporter Assay System (Promega, 0000170721, USA), and Histostain-Plus Kit (R&D, USA).

### Primary nucleus pulposus monolayer culture and stimulation

All intervertebral disc tissues used in this study were discarded as medical waste and were not used in the patients’ treatment. The nucleus pulposus tissues were washed 3 times with phosphate-buffered saline (PBS; Gibco, Grand Island, NY), minced into small fragments and digested in 0.25% (w/v) trypsin (Gibco) and 0.2% (w/v) type Π collagenase (Gibco) and then placed in PBS for approximately 3 hours at 37 °C in a gyratory shaker. Cells were filtered through a 70 μm mesh filter (BD, Franklin Lakes, NJ). Primary nucleus pulposus cells were cultured with growth medium (Dulbecco’s Modified Eagle’s Medium and Ham’s F-12 Nutrient Mixture (DMEM-F12; Gibco), 20% (v/v) fetal bovine serum (FBS; Gibco), 50 U/mL penicillin, and 50 μg/mL streptomycin (Gibco) in 100-mm culture dishes in a 5% (v/v) CO_2_ incubator. The cells were passaged at approximately 80% (v/v) confluence using trypsin and subcultured in a 60-mm culture dish (2.5 × 10^5^ cells/well). Cells that had been passaged no more than twice were used in the subsequent experiments. For all cell stimulation experiments, 10^5^ cells were seeded in each well of 24-well plates and 106 cells were seeded in each well of 6-well plates. When cells were grown to 80% (v/v) confluence, the indicated doses of recombinant human IL-1β (R&D), TGF-β (R&D) or different inhibitors under concentrations without cytotoxicity were used to stimulate cells. 48 hours later, cells were collected for RNA isolation, western blot, or immunofluorescence.

### Rabbit intervertebral disc degeneration model

The intervertebral disc degeneration model of New Zealand rabbits was established using defined needle gauges and depths^12^. 27 New Zealand white rabbits, weighing approximately 3.0 kg, were used in this study. The rabbits were anesthetized with 10% (w/v) chloral hydrate (5 ml/kg) through the ear vein, the rabbits were then placed into a left lateral prone position, and a 20 cm × 16 cm preoperative preparation of skin was made. We made a longitudinal incision approximately 10 cm from the iliac crest to the 12th rib margin, then discovered the gap between the abdominal external oblique and back muscles, and entered through an extraperitoneal approach. According to the location of the iliac crest, which is parallel to the L6 vertebrae, we exposed the anterior surfaces of 3 consecutive lumbar IVDs (L2–L3 to L4–L5) using a 21 G puncture needle to puncture the intervertebral disc, and the depth of the needle stab was controlled at exactly 5 mm by a hand-made stopper. The needle was rotated 360° and withdrawn. The wound was then thoroughly irrigated with sterile saline and closed with layered sutures. The rabbits were returned to their cages after a short recovery observation and mobilized *ad lib*. MRIs of the lumbar spine were repeated at 4-, 8-, and 12-weeks after surgery. The rabbits were killed at 4-, 8-, and 12-weeks post-surgery by injecting air, and the intact spinal columns were harvested for histological analyses.

### Luciferase reporter assay

The −2235~270 bp region was selected as the asporin promoter region. The promoter region was analyzed using the JASPAR core database, which revealed the presence of two putative p65 binding sites at −1743/−1733 and −41/−31 bp relative to the transcription start site. Then, an asporin reporter (−2235~270 bp, including the WT and mutants) was directly cloned into a pGL3 luciferase vector. Schematics of the wild-type and mutant human asporin promoter constructs were as follows: mutant 1, bp −1743 to bp −1733, TGGGATTTCTT to AGGGTTTACTT; mutant 2, bp −41 to bp −31, TGTGTTTTCCA to TGAGTATTGCA. Primers used for amplifying human asporin WT and mutated promoters with mutated binding sites underlined by polymerase chain reaction (PCR) were as follows: WT, forward: 5-CTGGCCTAACTGGCCGGTACCAGGCAGGGTTTACTTGATTGGGGTCATTTTGTAAAAAT-3, reverse: 5-CCAGATCTTGATATCCTCGAGCTGTCAGAAGAGAGTAGTCCTCCT-3; mutant 1, bp −1743 to bp −1733, forward: 5-GGATGTATGAGAATGGAGTGTGAGGCAGGGTTTACTTGATTGGGGTCATTTTGTAAAAA-3, reverse: 5-TTTTTACAAAATGACCCCAATCAAGTAAACCCTGCCTCACACTCCATTCTCATACATCC-3; mutant 2, bp −41 to bp −31, forward: 5-GTCTTGGCTACGATACAAACAGTGATGCAATACTCATGAGCAGTAACAAGTTTTAAT-3, reverse: 5-ATTAAAACTTGTTACTGCTCATGAGTATTGCATCACTGTTTGTATCGTAGCCAAGAC-3. Human primary nucleus pulposus cells were seeded into 24-well plates that were co-transfected with different plasmids (firefly reporter constructs containing the WT or mutant asporin promoter, a Renilla-expressing plasmid, a p65 plasmid or a control plasmid). Firefly and Renilla luciferase activities were measured 24 hours post-transfection using a Dual Luciferase Assay System (Promega). Firefly luciferase activity was normalized to Renilla luciferase activity.

### p65 overexpression

Total RNA from human nucleus pulposus cells was isolated using the traditional phenol-chloroform method following Trizol (15596-026, Invitrogen, USA) lysis. The cDNA was prepared by reverse transcription using a random primer (D3801, TaKaRa, DaLian, China) at 37 °C for 1 hour after the denaturation of total RNA. The full coding region of the p65 gene was amplified with polymerase chain reaction (PCR) as follows: denaturation at 95 °C for 5 minutes; 36 cycles of 95 °C for 30 sec, 52 °C for 30 sec and 72 °C for 90 sec; and post-elongation for 10 minutes at 72 °C. The PCR mixture was composed of 5 µl 10 × PCR buffer, 0.2 mM dNTPs, 0.2 µM of each primer of p65-F (GAGGATCCCCGGGTACCGGTCGCCACCATGGCGGAGCCGAGCGGC)/p65-R (TCACCATGGTGGCGACCGGGCTGACACTCAACTGAGCA), 2 U PFU polymerase (101060002, HarO, Shanghai, China) and sterile distilled water. The human p65 gene was then subcloned into lentiviral vector pGV208 (GV208, Genechem, Shanghai, China) using T4 ligase (EL0011, Fermentas, Burlington, Canada), followed by transformation with *E. coli* DH5α competent cells (C502-03, Vazyme, Nanjing, China). The p65 overexpression plasmid was transfected into human nucleus pulposus with Lipofectamine 3000 (Life technologies), and the over-expression efficiency of p65 was tested by western blot.

### Asporin overexpression

Total RNA from human nucleus pulposus cells was isolated using the traditional phenol-chloroform method following Trizol (15596-026, Invitrogen, USA) lysis. The cDNA was prepared by reverse transcription using random primer (D3801, TaKaRa, DaLian, China) at 37 °C for 1 hour after the denaturation of total RNA. The coding region of the human asporin gene from the D14 allele was obtained by PCR with the primer pair (forward: GAGGATCCCCGGGTACCGGTCGCCACCATGGCGGAGCCGAGCGGC, reverse: TCACCATGGTGGCGACCGGGCTGACACTCAACTGAGCA) and cloned into the pGV208 (GV208, Genechem, Shanghai, China) vector, followed by the transformation into *E. coli* DH5α competent cells (C502-03, Vazyme, Nanjing, China). The asporin overexpression plasmid was transfected into human nucleus pulposus cells with Lipofectamine 3000 (Life technologies), and the overexpression efficiency was tested by western blot.

### shRNA preparation and targeting gene knockdown

For gene silencing, human asporin- and p65-specific shRNAs (short hairpin RNAs; Supplementary Table 3) were cloned into the pLL3.7 vector (Addgene) as the manufacturer described. Four micrograms of pLL3.7 constructs containing specific shRNAs, 4 mg of packaging plasmid psPAX2 (Addgene) and 2 mg of envelope plasmid pMD2G (Addgene) were used to transfect human embryonic kidney 293 T cells via the calcium phosphate precipitation method. Forty-eight hours later, lentiviruses containing target gene shRNA were collected and used to transfect primary nucleus pulposus cells. The blockage efficiency of shRNA was tested by either western blot or PCR with reverse transcription (RT–PCR).

### Western blot analysis

The intervertebral disc tissues or the nucleus pulposus cells with different treatments were lysed in radioimmunoprecipitation assay (RIPA) buffer and supplemented with phosphatase inhibitors and protease inhibitor cocktail (Biotech Well, Shanghai, China). Lysates were centrifuged for 20 minutes at 12,000 × g. The protein concentration was determined using a BCA protein assay (Beyotime, Jiangsu, China), and equivalent amounts of protein (20 μg) were separated via sodium dodecyl sulfate polyacrylamide gel electrophoresis and transferred onto polyvinylidene fluoride membranes after being blocked in Tris-buffered saline Tween-20 with 5% (w/v) milk powder (2 hours) and then incubated with a specific antibody for asporin (rabbit anti-human, Abcam, ab58741, USA; rabbit anti-rabbit, Sigma, AV42487, USA), p65 (rabbit anti-human, Abcam, ab7970, USA), Aggrecan (SANTA CRUZ, sc-25674, USA), collagen Π (Abcam, ab3029, USA) and GAPDH (Abcam, ab181602, USA) at 4 C° with gentle shaking overnight. After washing, the membranes were incubated with the respective secondary antibody (Jackson, USA) at the appropriate concentration for 2 hours at room temperature. Immunolabeling was performed using enhanced chemiluminescence reagents (Amersham Biosciences, Roosendaal, The Netherlands). Signal intensities were quantified using ImageJ software.

Uncropped images of the blots for Figs 1b,e, 2b, 3d–f, 5e,h and 6e,h are shown in supplementary Figure 3.

### Immunohistochemistry

Decalcified intervertebral disc sections were boiled in 10 mM sodium citrate (pH 6.0) for 5 miniutes to retrieve antigens. Sections were quenched with 3% (v/v) hydrogen peroxide for 15 miniutes to reduce endogenous peroxidase activity and blocked with 3% (w/v) normal goat serum in Tris-buffered saline. The sections were then incubated with asporin (rabbit anti-human, Abcam, ab58741, USA; rabbit anti-rabbit, Sigma, AV42487, USA) or goat IgG as a control at 4 °C overnight, followed by biotinylated secondary antibodies and development using a peroxidase-labeled streptavidin–biotin staining technique (DAB kit, Invitrogen). Nuclei were counterstained with hemalum (FARCO Chemical Supplies, Hong Kong). The slides were visualized with a microscope (ZEISS, AXIO).

### Immunofluorescence staining

Primary nucleus pulposus cells that had undergone different treatments were used for immunofluorescence staining, and 2% (v/v) PFA was used to fix the samples. After 10 miniutes of fixation and subsequent pretreatment with antigen retrieval solution, the cells were stained with p65 and asporin antibodies for 1 hour at room temperature. Then, the cells were reprobed with Alexa Fluor® 488-conjugated goat anti-rabbit (Life, 1583138, USA) and then mounted in ProLong Gold antifade reagent with DAPI (Life, 1683678, USA) and visualized using a laser scanning confocal microscope (Olympus Fluoview, Japan).

### Statistical analysis

All data are present as mean ± SEM. We did analyses of multiple groups by one-way or two-way ANOVA with Bonferroni post test of GraphPad prism version 5. For all statistical tests, we considered *P* value < 0.05 to be statistically significant.

## Electronic supplementary material

Supplementary figure
