# Supplementary material for: IL-1β increases asporin expression via the NF-κB p65 pathway in nucleus pulposus cells during intervertebral disc degeneration
Source: Sci Rep. 2017 Jun 23;7:4112. doi: 10.1038/s41598-017-04384-3 (PMC5482889; doi:10.1038/s41598-017-04384-3)
Supplement: Supplementary file 1 — Supplementary figure [file 41598_2017_4384_MOESM1_ESM.pdf]

IL-1β increases asporin expression via the NF-κB p65 pathway in nucleus pulposus cells during intervertebral disc degeneration

Shengjie Wang [1†]; Chao Liu [1†]; Zhongyi Sun [3†]; Peng Yan [1]; He Liang [3]; Kai Huang [1]; Changwei Li [2*]; Jiwei Tian[1*]

[1] Department of Orthopedics, Shanghai First People's Hospital Affiliated to Shanghai Jiao Tong University, School of Medicine, 100, Haining Road, Shanghai,200080, People's Republic of China.

[2] Shanghai Key Laboratory for Prevention and Treatment of Bone and Joint Diseases with Integrated Chinese-Western Medicine, Shanghai Institute of Traumatology and Orthopedics, Ruijin Hospital, Shanghai Jiaotong University School of Medicine, 197 Ruijin 2nd Road, Shanghai, 200025, People's Republic of China.

[3] Department of Orthopedics, Songjiang District Central Hospital of Shanghai， 746, middle-Zhongshan Road, Shanghai,201600, People's Republic of China.

† These authors contributed equally to this work

*** Corresponding author:**

Changwei Li

Shanghai Key Laboratory for Prevention and Treatment of Bone and Joint

Diseases with Integrated Chinese-Western Medicine, Shanghai Institute of Traumatology and Orthopedics, Ruijin Hospital, Shanghai Jiaotong University School of Medicine, Shanghai, 200025, China; Tel: +86-21-64313534, Fax: 011-86-21-64335742; Email: changwei393331@163.com

Jiwei Tian

Department of Orthopedics, Shanghai First People's Hospital Affiliated to Shanghai Jiao Tong University, School of Medicine, 650, New Songjiang Road, Songjiang District, Shanghai, 201620, China; Tel: +86-21-37798591; Fax: +86-21-37798833; Email: tjw609@163.com

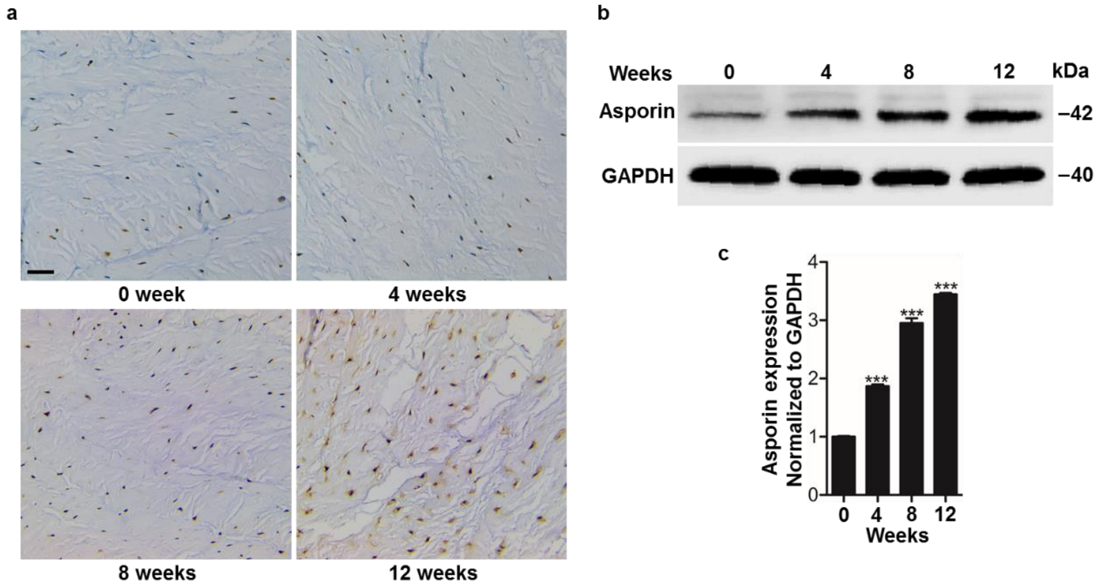

Supplementary figure 1. Needle puncture injury induced time-dependent expression of asporin in the annulus fibrosus and cartilage endplate. (a) Immunohistochemical staining of asporin in rabbit annulus fibrosus after 0, 4, 8 and 12 weeks of needle puncture injury. Scale bars represent 10 μm. (b & c) Western blot analysis of asporin in rabbit cartilage endplate after 0, 4, 8 and 12 weeks of needle puncture injury. ****P<0.001,* *P*-values were analyzed by one-way ANOVA.

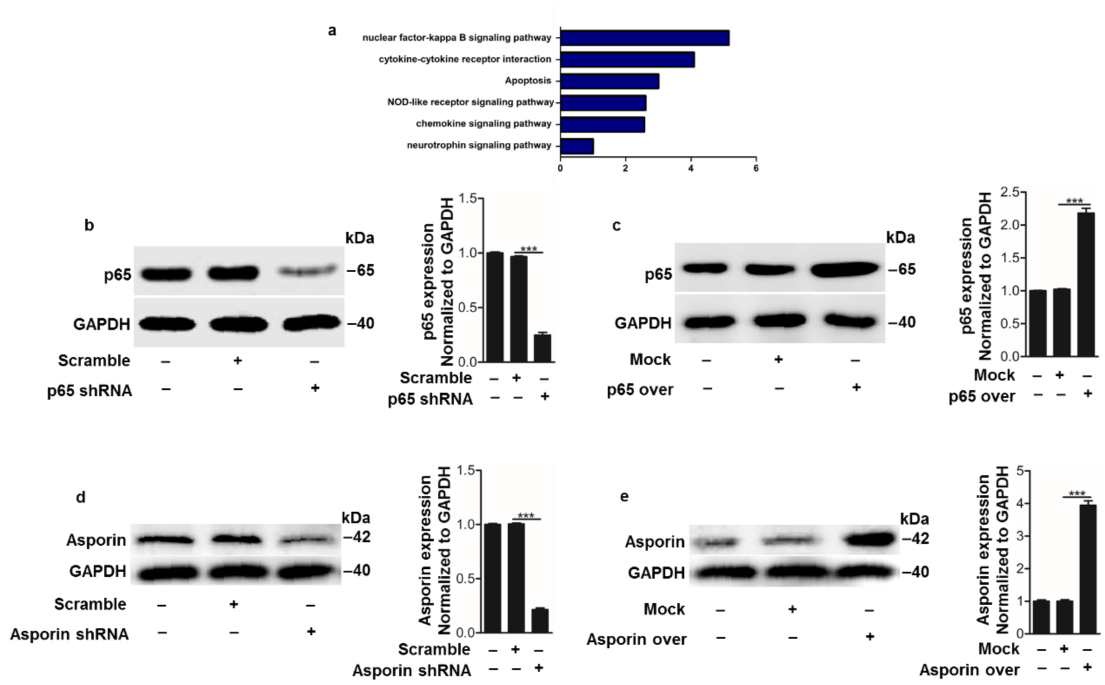

Supplementary figure 2. (a) The comparison of genes expression between degenerated discs and non-degenerated discs. (b) p65 expression in nucleus pulposus in the presence or absence of p65 shRNA. (c) p65 expression in nucleus pulposus after transfected with or without p65 overexpression plasmid. (d) Asporin expression in nucleus pulposus in the presence or absence of asporin shRNA. (e) Asporin expression in nucleus pulposus after transfected with or without asporin overexpression plasmid. ****P<0.001, P*-values were analyzed by one-way ANOVA.

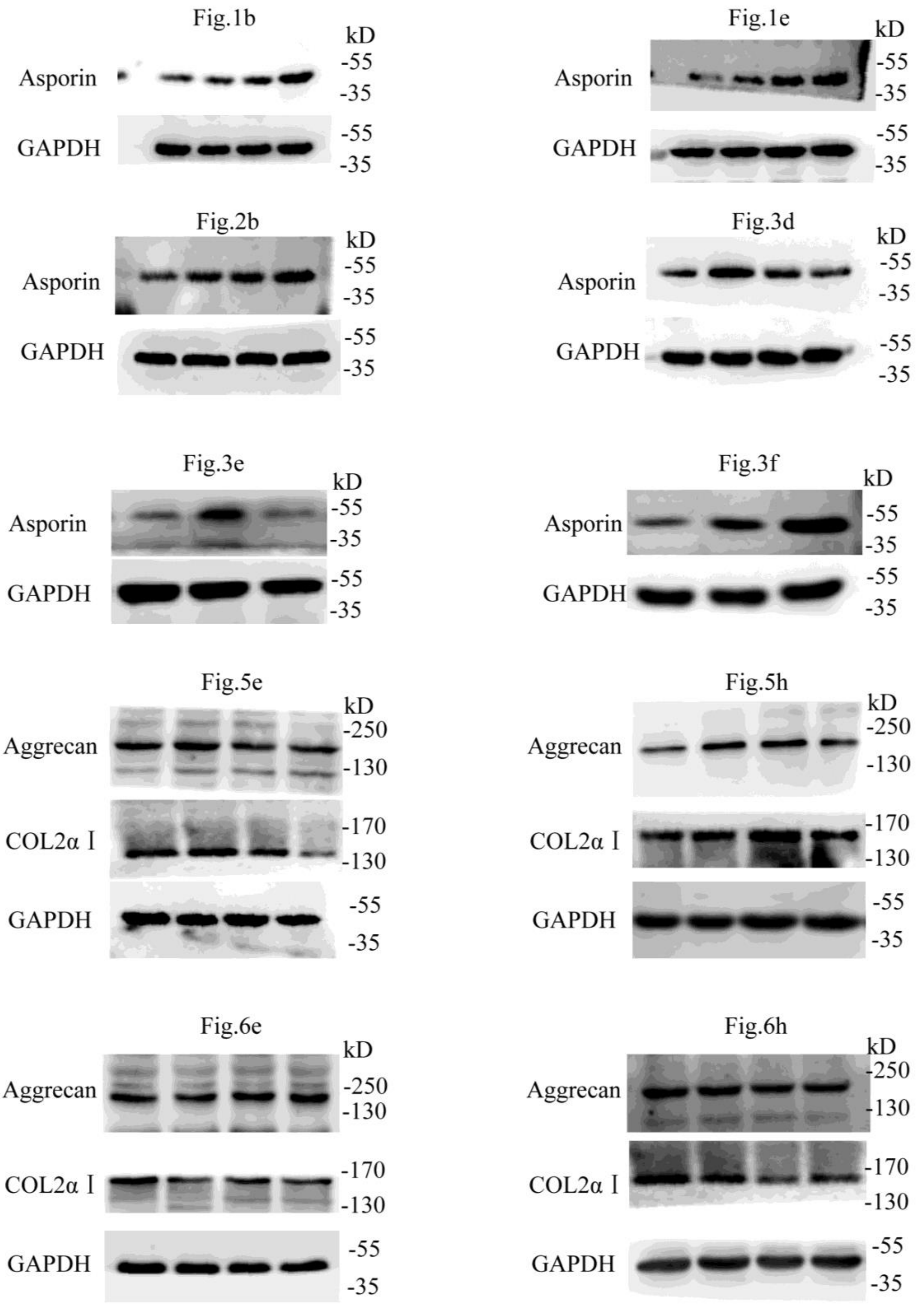

Supplementary figure 3. Original immunoblots for indicated figures.
